# Stability Control and Turning Algorithm of an Alpine Skiing Robot

**DOI:** 10.3390/s19173664

**Published:** 2019-08-23

**Authors:** Si-Hyun Kim, Bumjoo Lee, Young-Dae Hong

**Affiliations:** 1 Department of Electrical and Computer Engineering, Ajou University, Suwon 16499, Korea; 2 Department of Electrical Engineering, Myongji University, Yongin 17058, Korea

**Keywords:** navigation based on LiDAR sensor, skiing robot, stability control, turn radius

## Abstract

This paper proposes a general stability control method that uses the concept of zero-moment-point (ZMP) and a turning algorithm with a light detection and ranging (LiDAR) sensor for a bipedal alpine skiing robot. There is no elaborate simulator for skiing robots since the snow has complicated characteristics, such as compression and melting. However, real experiments are laborious because of the many varied skiing conditions. The proposed skiing simulator could be used, so that a humanoid robot can track its desired turning radius by modeled forces that are similar to real ones in the snow. Subsequently, the robot will be able to pass through gates with LiDAR sensors. By using ZMP control, the robot can avoid falling down while tracking its desired path. The performance of the proposed stabilization method and autonomous turning algorithm are verified by a dynamics simulation software, Webots, and the simulation results are obtained while using the small humanoid robot platform DARwIn-OP.

## 1. Introduction

Research into humanoid robots is one of the most exciting topics in the field of robotics. As proof, robot competitions, such as the DARPA Robotics Challenge (DRC), are becoming increasingly popular. In particular, the Ski Robot Challenge was held for the first time during the PyeongChang Winter Olympic Games. From this perspective, the importance of skiing robot is increasing.

Humanoid robots are those with two legs and there have been many developments in their walking abilities over the last decade. In particular, the concept of the linear inverted pendulum model (LIPM) is widely used [1,2]. It is advantageous that dynamic calculation is easy because the vertical component of the center of gravity is always constant, which decouples the sagittal and lateral components of the center of gravity. The zero-moment point (ZMP) is another concept [3]. It is possible for a robot to maintain stability by moving its center of mass (CoM) to control the ZMP. These concepts are also useful for skiing robots.

The objective of skiing is for the skier to reach their destination. Good skiers tilt their body to change direction. The relationship between tilting angle and turning radius, and the forces acting on a skier while skiing are already known [4,5,6,7,8]. Using this relationship, there have been a few attempts to experiment with skiing robots in real environments. Yoneyama et al. tested a skiing robot that had legs with six degrees of freedom, like those of human athletes on an artificial grass slope [9]. By considering the relationship between joint motions, the skiing robot could turn on the slope by abduction–adduction motion. Iverach-Brereton et al. used a humanoid robot to verify their braking motion and the performance of posture control in a snowy environment while using proportional-integral-derivative (PID) controllers [10]. In addition, ZMP has been applied to maintain stability during skiing and realize the navigation algorithm while using cameras [11,12,13,14]. Almost all of them carried out an experiment in a real environment; however, skiing experiments are very challenging tasks, because snow requires a wide variety of conditions, such as temperature or humidity. Unfortunately, there are no simulation tools that can realize the precise properties of snow. Roller skis were used to test the skiing robot in a real environment to overcome this problem, but the experiments with roller-skis did not work well, because roller skis with wheels are not suitable for carving turns [10].

This paper presents a simulator that can test a skiing robot by applying modeled forces that are similar to real forces when in snow. When considering the relationship between the leaning angle and turning radius, a skiing robot can track its desired path by leaning its body in the simulation. In addition, a novel navigation algorithm has been proposed that uses a light detection and a ranging (LiDAR) sensor. ZMP control is applied to avoid falling over while tracking the desired path. Finally, this method’s performance is verified with the proposed simulator.

The paper is organized, as follows: Section 2 describes the system overview. First, the robot kinematics of what we used is shown. Next, the turn radius of the carving turn is described and the relationship between the ski’s edging angle and the robot’s center of mass (CoM) is derived. Finally, the process of modeling the forces on the ski plate that results from the ski–snow interaction is explained. Section 3 proposes a turning algorithm while using a LiDAR sensor. Section 4 describes the stability control using the ZMP control. The ZMP is reviewed and the method of applying the ZMP to a skiing robot is described. Section 5 presents the simulation results and Section 6 discusses the results in comparison with previous studies. Finally, Section 7 concludes the paper with possibilities of development and future works with the proposed framework.

## 2. System Modeling Consideration

The proposed framework is composed of four parts. First, the relationship between the robot’s leaning angle and the turning radius is modeled by approximation. Second, the modeling procedure for the forces from the snow to ski plate is introduced. Third, an autonomous navigation algorithm for a skiing robot is proposed. Finally, the method by which the robot maintains stability is explained.

### 2.1. Robot Kinematics

**Figure 1:**
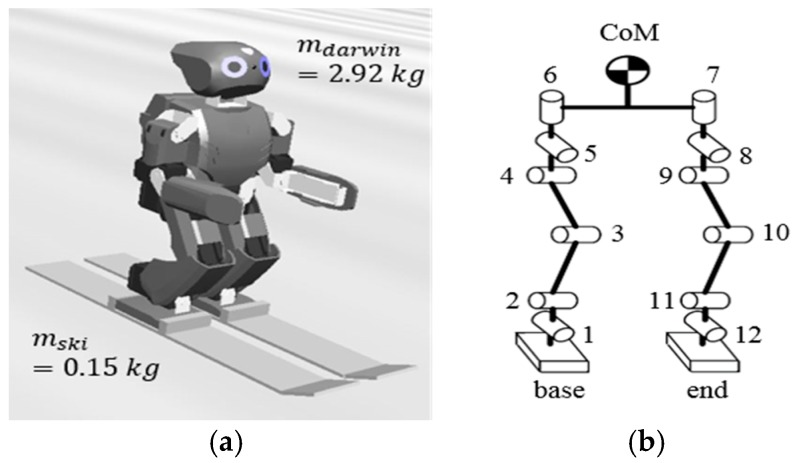
(**a**) DARwIn-OP robot and ski; (**b**) Kinematic lower body configuration of DARwIn-OP.

The DARwIn-OP, as shown in Figure 1, was used in the skiing simulation. The kinematics are solved by homogeneous transformation matrices from the base, which means the supporting foot (right foot) to the CoM and to the end of the link (left foot). Each leg of the robot consists of six motors. Therefore, rotation matrices from the supporting foot to the CoM and to the end of the link are as followed:

(1) $$R_{1}^{CoM}=\prod_{i=1}^{5}R_{i}^{i+1}\;,\;R_{1}^{end}=\prod_{i=1}^{11}R_{i}^{i+1}$$

where $R_{1}^{CoM}$ and $R_{1}^{end}$ are the rotation matrices from the base to the CoM and to the end of the link and $R_{i}^{i+1}$ is a rotation matrix from the frame {i} to the frame {i+1}. In this paper, the navigation algorithm and stability control are performed by changing the position of the CoM. The inverse kinematics are solved by an iterative method using Jacobian transpose matrices with roll–pitch–yaw (RPY) conventions to place the CoM at the desired position and the end of the link on the ground.

(2) $${\dot{x}}_{1}^{CoM}=J_{1}^{CoM}\dot{\theta }\;,\;{\dot{x}}_{1}^{end}=J_{1}^{end}\dot{\theta }$$

where $x_{1}^{CoM}$ is a 6 × 1 matrix that is composed of positions and orientations that were obtained by Equation (1), θ is a 6 × 1 or 12 × 1 matrix composed of joint angles, $J_{1}^{CoM}$ is a 6 × 6 matrix, and $J_{1}^{end}$ is a 6 × 12 matrix. The angles in the robot’s joints are calculated by using the transpose of Equation (2), which is the pseudo-inverse iterative approach to solving inverse kinematics.

### 2.2. Turn Radius of a Carving Turn

**Figure 2:**
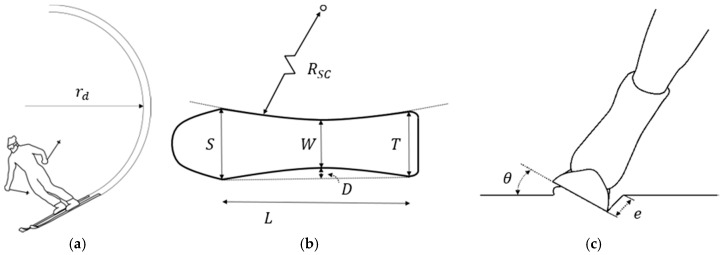
(**a**) Turn radius $r_{d}$; (**b**) Parameters characterizing of ski geometry; (**c**) Edging angle θ and penetration depth e.

A carving turn is a turning technique where the ski shifts to one side on its edges. Shaped skis make carved turns possible with a short turn radius. The turn radius of a carving turn, as follows [5]:

(3) $$r_{d}=\frac{R_{SC}\;cos\theta }{1+\left(e/D\right)\;sin\theta \;cos\theta }$$

where $r_{d}$ is the turn radius, $R_{SC}$ is the sidecut radius of the ski plate, θ is the edging angle, e is penetration depth, and D is the side cut of the ski plate; these are shown in Figure 2. In [5], they used a ski of mass 3.8 kg and of sidecut radius 15.4 m, the mass of the skier was 80 kg, and the mass of the DARwIn-OP robot we used was 2.922 kg. Therefore, the mass of the ski was proportionally set to 0.15 kg. Since the maximum value of cosθ is 1, $R_{SC}$ was set to 22 m to test a variety of turn radii. Additionally, the shovel (front of the ski) and tail (back of the ski) are both 70 mm and the length of the ski L is 500 mm. This makes the waist (middle of the ski) 67 mm. Thus, the side cut D of the ski is 1.5 mm.

Snow is divided into hard snow and soft snow, depending on hardness. The difference between the two is that penetration does not occur in the case of the hard snow, but it occurs in the case of the soft snow. However, penetration is difficult to measure or predict on a ski slope. Therefore, in the simulation, we used the turn radius formula of the hard snow. If the penetration e is known, more accurate simulation results can be obtained by using Equation (3). The turn radius formula in the hard snow that is the same as when e=0 in the turn radius equation of the soft snow is as follows.

(4) $$r_{d}=R_{SC}\;cos\theta$$

There are several types of skis, including traditional skis and carving skis. The main difference between the two is the sidecut radius. Traditional skis have large sidecut radii, while the carving skis have small sidecut radii. Additionally, unlike downhill skis with high bending stiffness, carving skis are designed to bend well. The carving ski basically does not need skidding when turning [11]. That is, carving skis exploits their shape to make turns. Because of this, the sidecut radius of the ski becomes an important factor in determining the turn radius, as in Equation (3). Although carving skiing essentially does not require skidding to turn, skidding always occurs somewhat in real snow environments, because snow has limited strength [8]. Therefore, Equation (3) is the lower bound of the actual turn radius, and the turn radius in the real snow can be larger than that in the simulation.

**Figure 3:**
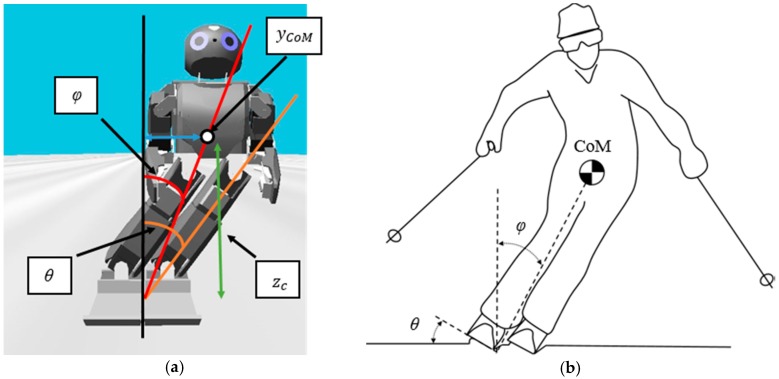
(**a**) φ is the lean angle between $y_{CoM}$ and middle of the vertical line, θ is an edging angle, $z_{c}$ is a constant CoM height normal to the snow surface; (**b**) Approximation of φ and θ.

Since a 3-D LIPM was used, there is a constant CoM height $z_{c}$ normal to the snow surface. Using this leads to the following geometrical equation.

(5) $$\varphi =\tan^{-1}\left(\frac{y_{CoM}}{z_{c}}\right)$$

where φ is the lean angle between CoM and the middle of the vertical line and $y_{CoM}$ is the distance between the lateral CoM and the middle of the vertical line. In Figure 3b, the edging angle θ was almost the same as φ. Therefore, if θ and φ are equal through an approximation, we can obtain the equation for $y_{CoM}$ and $r_{d}$ by combining Equations (4) and (5), as follows.

(6) $$\theta =\cos^{-1}\left(\frac{r_{d}}{R_{SC}}\right)\approx \varphi =\tan^{-1}\left(\frac{y_{CoM}}{z_{c}}\right)$$

(7) $$y_{CoM}=z_{c}\tan\left(\cos^{-1}\left(\frac{r_{d}}{R_{SC}}\right)\right)$$

This equation means that, if the desired turn radius $r_{d}$ is selected, the robot will change its $y_{CoM}$ according to Equation (7). Figure 3a shows a representation of the relationship between the parameters and Figure 3b shows an approximation between φ and θ.

### 2.3. Modeling the Forces Applied to the Ski Plate

This paper’s purpose is to enable the simulation of a skiing robot’s carving turn in a simulator. However, it is difficult to realize the properties of the real snow in a simulator. Therefore, most of the researchers have conducted real experiments to try to verify their algorithms [9,10,11]. Some researchers said that there is no simulation tool that can accurately reflect the models of the skiing robot, the ski, and the terrain in real-time. Therefore, they developed their own system and conducted the simulation. However, the turn radius of the skiing robot in the virtual environment was controlled by a joystick. It was stated that, in the future, they wish to replace the human operator, with a high-level control algorithm to achieve autonomous behavior [13]. In this paper, the simulator that applies the modeled force from the snow to the ski plate is proposed. In particular, our simulator is automated and it does not require a joystick.

Many researchers have already studied the forces that are applied to skis during a carving turn. The force equation is as follows [6].

(8) $$F_{tl}=F_{C}+F_{lat}=\frac{mv^{2}}{r_{d}}\mp W\;\sin\alpha \;\cos\beta$$

where $F_{tl}$ is the total radial force, $F_{C}$ is the centrifugal inertial force, $F_{lat}$ is the lateral gravitational force, m is the total mass of the skier with skis, v is the skier’s linear velocity, *W* is the gravitational force, α is the slope inclination, and β is the angle between the horizontal direction of the slope and the ski. $F_{C}$ can be rewritten, as follows:

(9) $$F_{C}=\frac{mv^{2}}{r_{d}}=\frac{mv^{2}}{R_{SC}\;cos\theta }$$

**Figure 4:**
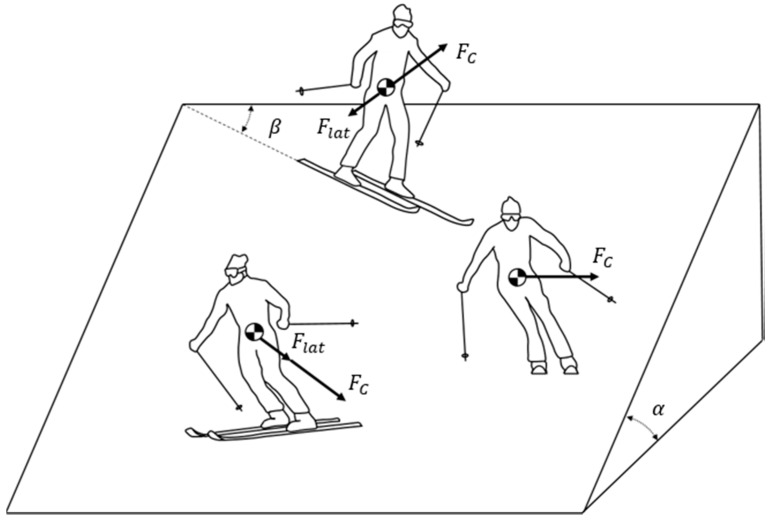
The diagram of the lateral forces that act on a skier.

This force can be applied to both hard snow and soft snow. The reason is that when the type of snow changes, altered $r_{d}$ affects the value of $F_{C}$. Additionally, since the skidding is minimized in the carving turn, $F_{C}$ is similar to the force that is generated in the actual snow environment. However, $F_{C}$ is only valid in real snow conditions. Therefore, we applied modeled forces the same as $F_{C}$ to the middle of the ski plates in the simulator instead of realizing real snow conditions. Thus, it was possible to simulate a carving turn like a real experiment on a flat solid slope in the simulator. Figure 4 is a diagram of $F_{C}$ and $F_{lat}$.

## 3. Navigation Using a LiDAR Sensor

Alpine skiing is a sport that slides down snowy slopes while using skis with a fixed heel binding. There are four categories of official alpine ski competitions: slalom, giant slalom, super giant slalom, and downhill. The primary goal of these is to get through the designated gates (a set of two flags) as quickly as possible. Therefore, gate tracking capability and fast turning techniques were needed. However, navigating during skiing is very hard, even for human skiers, because it is difficult to control velocity. Therefore, the skiing robot needs to be able to find the target gates, reduce the current speed, or generate the desired turn radius that is appropriate for the current speed, and take motion to follow it. The proposed navigation algorithm for a skiing robot to track the target gates is realized by using a LiDAR sensor.

### 3.1. LiDAR Sensor

LiDAR sensors can tell the distance from and direction to an object. In [11], gates were recognized through image processing while using a camera. However, since cameras are sensitive to light, a target may be unrecognized, depending on the environment. Meanwhile, a LiDAR sensor is accurate in terms of distance and direction, irrespective of light, and this is advantageous in terms of calculation amount, because there is no need to perform image processing. The LiDAR sensor that we used scanned the area 180° ahead in one dimension and the resolution was 0.25°. The maximum scannable distance was 80 m.

### 3.2. Navigation Algorithm

**Figure 5:**
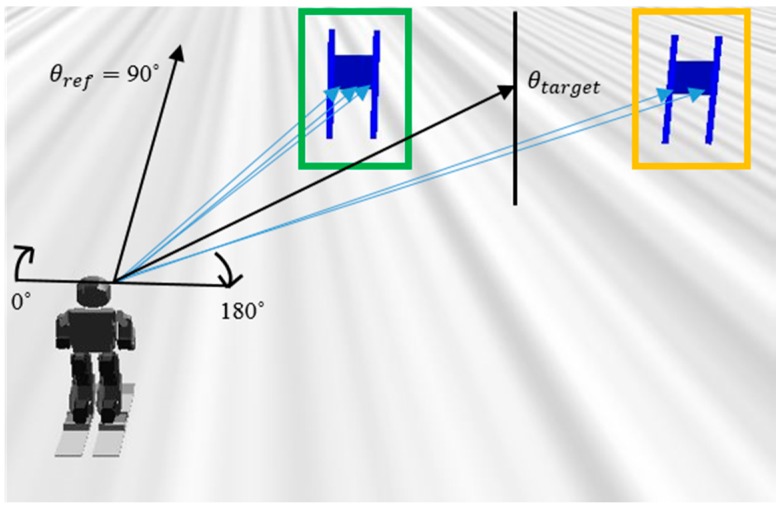
$\theta_{ref}$ is the direction of the skiing robot and $\theta_{target}$ is the angle from the skiing robot to the middle of the gate.

The direction in which the robot looks is 90° in the LiDAR sensor. Therefore, if the robot’s motion is controlled, such that the direction of the gate obtained through the LiDAR sensor is maintained at 90°, the robot can track the target gate. The calculation method for the direction information of the gate that is used to generate the control input and move to the center of the gate is as follows.

(10) $$\theta_{target}=\;\left(\frac{\sum_{k=1}^{i}\theta_{left,k}}{i}+\frac{\sum_{k=1}^{j}\theta_{right,k}}{j}\right)/2$$

where $\theta_{target}$ is the angle from the front of the skiing robot to the middle of the target gate, $\theta_{left}$ and $\theta_{right}$ are the angles to the left flag and to the right flag, and i and j are the numbers that are detected by the LiDAR sensor in the left flag and the right flag. For example, in Figure 5, the LiDAR sensor detected the left flag (green box) three times and detected the right flag (yellow box) two times. Subsequently, $\theta_{target}$ was calculated using the angles and the number of detections.

Through this, we can derive the control law to satisfy the desired turn radius, as follows.

(11) $$u_{r_{d}}=K_{P,r_{d}}\;e_{r_{d}}+K_{D,r_{d}}\;{\dot{e}}_{r_{d}}$$

where $u_{r_{d}}$ is the control input of the turn radius controller, $K_{P,r_{d}}$ is the proportional gain of the turn radius controller, $K_{D,r_{d}}$ is the derivative gain of the turn radius controller, and $e_{r_{d}}$ is $\left(\theta_{ref}-\theta_{target}\right)$. $\theta_{ref}$ is 90°, because the purpose of the turn radius control is to direct the robot to the target gate, and the robot’s orientation is 90° in the LiDAR sensor. Figure 5 depicts $\theta_{ref}$ and $\theta_{target}$.

The navigation algorithm is performed, as follows. $\theta_{target}$ is first obtained by the LiDAR sensor and $e_{r_{d}}$, the difference between $\theta_{ref}$ and $\theta_{target}$ is calculated. Afterwards, $e_{r_{d}}$ is used to get $u_{r_{d}}$ as in Equation (11), and $u_{r_{d}}$ is added to current $y_{CoM}$. At this time, the sum of $u_{r_{d}}$ and $y_{CoM}$ is named $y_{CoM,ref}$. When $y_{CoM}$ of the robot moves to $y_{CoM,ref}$, the lean angle changes accordingly. If the edging angle and the lean angle are approximated, the robot turns with the turn radius that was calculated by Equation (4). The advantage of this method is that the turn radius is changed in real-time so that the robot is towards the center of the gate, allowing the robot to track the gate regardless of the current speed or distance between the robot and the gate. In addition, the robot can pass through the target gate while using this real-time navigation algorithm, even if there is an error of turn radius due to skidding or modeling error.

However, if there are more than two gates within the maximum scannable distance and orientation, only the gates within 8 m will be recognized based on the nearest gate to the robot. In the giant slalom, which is a type of alpine skiing competition, the legal gate width is 4.5–8 m (min./max) and the distances between the gates are 15–27 m (min/max). Therefore, it is reasonable to set the searching range to 8 m. The skiing robot can ski in various gate environments while using this condition.

## 4. Stability Control

One of the most important priorities for skiing robots is skiing without falling over. Therefore, maintaining stability in robots is one of the most important purposes. Unlike walking robots, the sagittal side of the skiing robot is equipped with a ski, which makes it difficult to fall over. Therefore, the stability control in the lateral direction is key. The ZMP concept is used to maintain stability under dynamic conditions.

**Figure 6:**
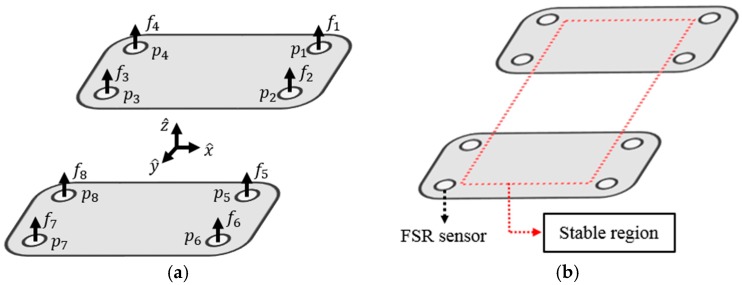
(**a**) Robot’s feet with force-sensing resistor (FSR) sensors; (**b**) Stable region of the zero-moment point (ZMP).

ZMP means that the sum of the moments acting on the robot’s feet is zero. The closer the ZMP to the center of the foot, the more stable it becomes. If the ZMP moves away from the robot’s support polygon, it will fall down. In addition, robots are generally modeled as LIPM, because dynamic computation becomes easier when the CoM height from the ground is constant. The ZMP equation of the LIPM can be expressed, as follows.

(12) $${\ddot{x}}_{c}=\frac{g}{z_{c}}\left(x_{c}-p\right)$$

where $x_{c}$ is the position vector of the CoM with respect to the support origin, p is the ZMP vector with respect to the origin of the support polygon, and g is the gravity constant. The method of measuring the ZMP in a real robot can be obtained while using force-sensing resistor (FSR) sensors. The FSR sensor is a pressure sensor whose resistance changes when a force, pressure or mechanical stress is applied. It uses the property that the resistance value changes according to physical force or weight. Figure 6 shows the robot’s feet with FSR sensors. In Figure 6a, f denotes the sensed pressure and p denotes the position of the sensor. The ZMP is also known as the center of pressure. Therefore, the formula for obtaining the ZMP through FSR sensors is as follows.

(13) $$p_{x}=\frac{\sum_{i=1}^{8}p_{i,x}f_{i,x}}{\sum_{i=1}^{8}f_{i,x}},\;p_{y}=\frac{\sum_{i=1}^{8}p_{i,y}f_{i,y}}{\sum_{i=1}^{8}f_{i,y}}$$

In Figure 6b, the dotted red square means the stable region, and the robot will not fall if the ZMP is inside this region. The goal of stability control is to send the ZMP as far as possible to the middle of this stable region.

Using a LiDAR sensor allows for gate tracking and turning algorithms to be implemented. However, without considering the robot’s stability, it is possible to input a tiny turn radius that makes the centrifugal inertial force $F_{C}$ large according to Equation (9). In addition, according to Equation (4), the tiny turn radius makes the edging angle larger, which makes the robot’s ZMP move outward. Especially, when the turn radius very close to zero is calculated, the edging angle almost reaches 90°, and the robot can directly fall. Therefore, stability control is applied to minimize the robot’s instability due to excessive input while the robot tracks the gates. Stability control can move the ZMP towards zero and reduce the edging angle by slightly increasing the tiny turn radius.

**Figure 7:**
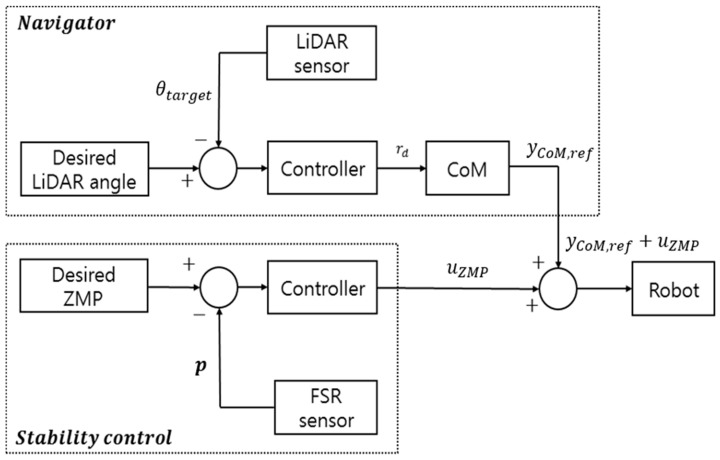
Overall navigation and stability control method of a skiing robot.

The control input of stability control is as follows:

(14) $$u_{ZMP}=K_{P,ZMP}\;e_{ZMP}+K_{D,ZMP}\;{\dot{e}}_{ZMP}$$

where $u_{ZMP}$ is the control input of the ZMP controller, $K_{P,ZMP}$ is the proportional gain of the ZMP controller, and $K_{D,ZMP}$ is a derivative gain of the ZMP controller, $e_{ZMP}$ is $\left(p_{y,ref}-p_{y}\right)$. $P_{y,ref}$ is a reference of the lateral side ZMP that is equal to zero. Thus, the input $y_{CoM}$ for the skiing robot changes, as follows.

(15) $$y_{CoM}=y_{CoM,ref}+u_{ZMP}$$

where $y_{CoM,ref}$ is the lateral CoM for meeting the nominal reference turn radius. If a fixed $y_{CoM}$ is applied, the robot could become stable again when the ZMP tries to move out of the supporting feet. Figure 7 shows the overall process.

## 5. Simulation Results

**Figure 8:**
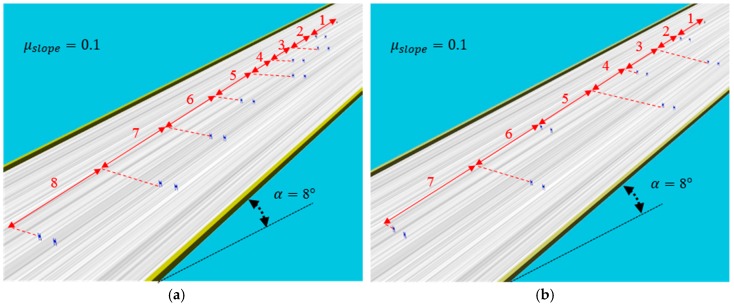
The ski slope and configuration of the gates (**a**) Short interval; (**b**) Long interval.

Simulations were performed by the dynamics simulation software, Webots [15]. Figure 8 shows the slope that we tested and the slope angle is 8°. The friction coefficient between the slope and ski plate is 0.1, which is the average for the actual ski slope. All of the gates were made of two flags, two meters apart. The differences between Figure 8a,b are the gaps between the gates. In Figure 8a,b, the gaps between gates are narrow and broad, respectively. Table 1 shows detailed distances of the gates.

**Figure 9:**
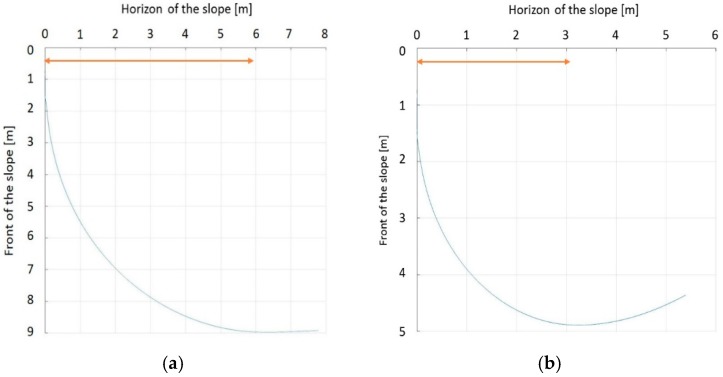
The skiing robot’s turn radius (**a**) $r_{d}=6$ [m] is inputted; (**b**) $r_{d}=3$ [m] is inputted.

First, we verified that the turn radius has been correctly implemented in the simulator. In Figure 9a,b, we can see the results of the trajectory when 3 m and 6 m are inputted to the robot, respectively. Although the robot cannot draw a complete circle because the velocity changes continuously as it moves down the slope, it can be seen that the turn radius comes close to 3 m and 6 m, respectively, through the graph.

**Figure 10:**
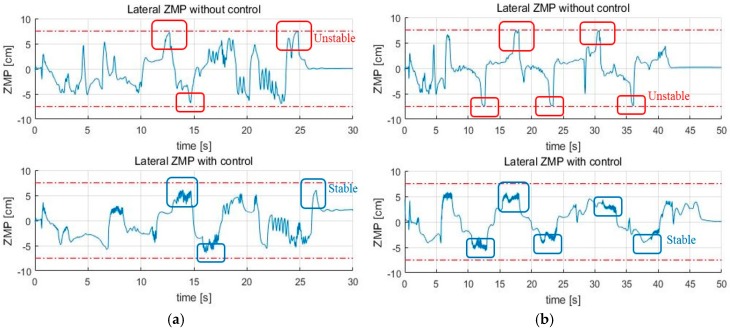
The results of the ZMP control (**a**) Short interval; (**b**) Long interval.

**Figure 11:**
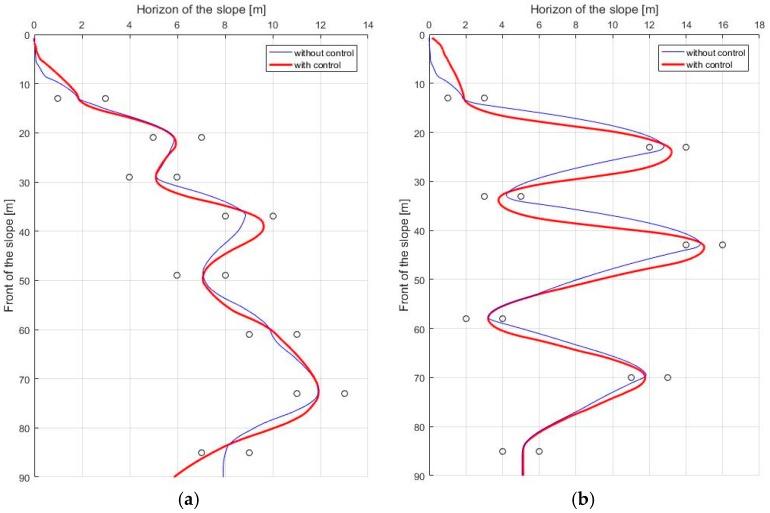
The trajectory of the skiing robot (**a**) Short interval; (**b**) Long interval.

Next are the results of the turn algorithm. In order for the skiing robot to track the target gate, the current linear velocity of the robot should not be too high. The reason is that the ZMP of the robot will saturate if the modeled force $F_{C}$ becomes too large. According to Equation (9), if the square of the linear velocity $v^{2}$ is increased, $r_{d}$ cannot be reduced by that much. The fact that $r_{d}$ cannot be reduced means that if $e_{r_{d}}$ is large, the robot cannot reach the target gate. On the other hand, even if the speed is moderate, if $r_{d}$ is calculated too small, the edging angle increases too much, and the ZMP can be saturated. In this situation, stability control is applied to solve this problem, and Figure 10 presents the results, which show that the ZMP is saturated at the tip of the feet before the control. However, ZMP moves toward 0 after the control, which shows that the stability is improved when compared to the conventional posture control method. Figure 11 shows the GPS trajectory before and after the control, respectively. In Figure 11, it seems to be that the trajectory of the robot without stability control (blue line) is more efficient than that of the robot with stability control (red line). The reason is that the robot turned to the lowest turn radius possible while only considering navigation. However, since the robot did not consider stability, even if the slope angle was slightly larger or the friction coefficient of the slope was slightly lower, the robot fell down when moving to the next gate.

**Figure 12:**
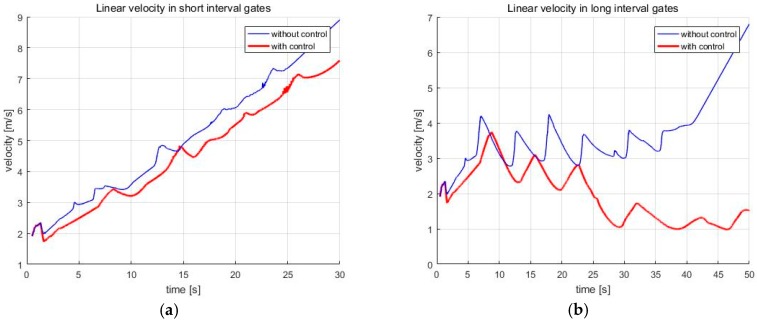
The linear velocity of the skiing robot (**a**) Short interval; (**b**) Long interval.

In addition, the speed of the skiing robot is reduced when it turns to pass through the gate. However, if the turn radius that is calculated by the navigation algorithm is too small, the skiing robot will turn too rapidly, which can make the robot unstable. At this time, when the stability control is applied, the turn radius is changed, so as not to be abrupt, so that the speed is further reduced than that before the control. These results can be seen in Figure 12. On the other hand, there is currently no way to control the speed in a simple sliding state. This is the subject of future research.

Next, the simulations were conducted with varying friction coefficients and slope angle in order to verify the performance of the navigation algorithm. As you can see in Figure 12, the skiing robot slows down during the turn. In real skiing, a skier can reduce the speed by attacking the skis relating to his moving direction. That is, shearing energy is dissipated, skidding occurs, and then speed is reduced. However, when the centrifugal force is large because of the small turn radius or high speed, attacking the skis could also happen, which can make skidding. Therefore, since the turn radius when the robot turns through the gate is much smaller than the turn radius when the robot moves to the gate, much more skidding occurs when turning. This is the reason that the skiing robot’s speed is reduced during the turn. In Figure 12, the speed of the skiing robot increases overall in short interval gates, while the speed decreases at the turning part in the long interval gates. This is because the turn radius for the robot to turn through the gate is smaller in the long interval gates than in short interval gates. Therefore, the effect of stability control increases in the long interval gates. Accordingly, we ran additional tests in the long interval gates.

**Figure 13:**
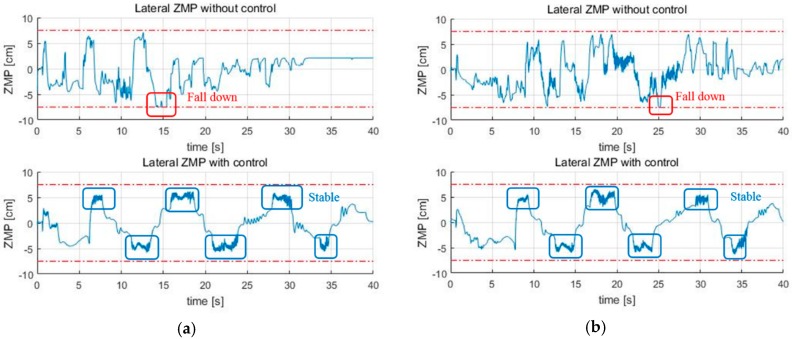
The results of the ZMP control in long interval gates (**a**) $\mu_{slope}=0.09$, $\alpha =8^{{}^{\circ}}$; (**b**) $\mu_{slope}=0.12,\;\alpha =10^{{}^{\circ}}$.

**Figure 14:**
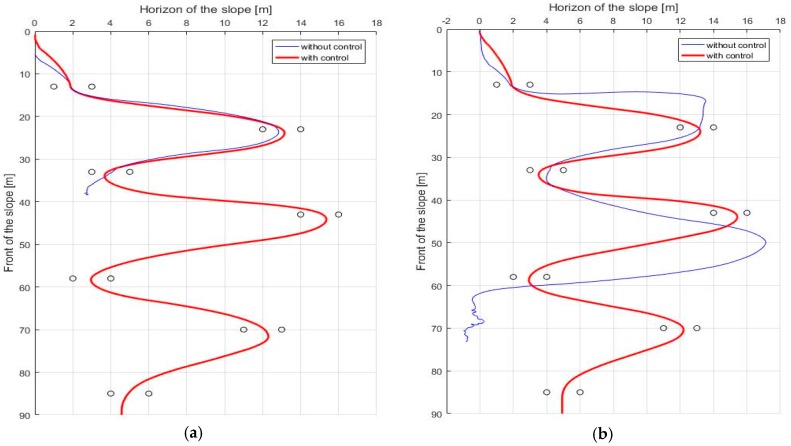
The trajectory of the skiing robot in long interval gates (**a**) $\mu_{slope}=0.09$, $\alpha =8^{{}^{\circ}}$; (**b**) $\mu_{slope}=0.12,\;\alpha =10^{{}^{\circ}}$.

**Figure 15:**
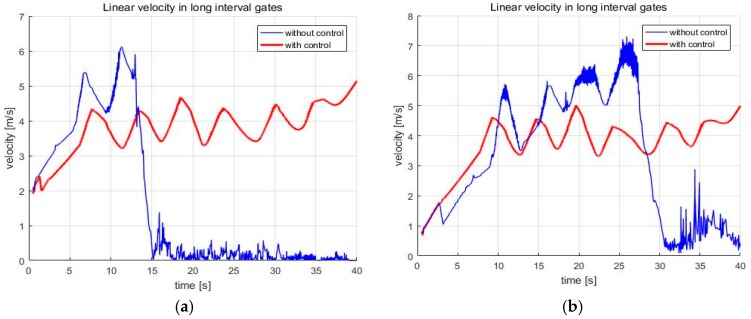
The linear velocity of the skiing robot (**a**) $\mu_{slope}=0.09$, $\alpha =8^{{}^{\circ}}$; (**b**) $\mu_{slope}=0.12,$ $\alpha =10^{{}^{\circ}}$.

**Figure 16:**
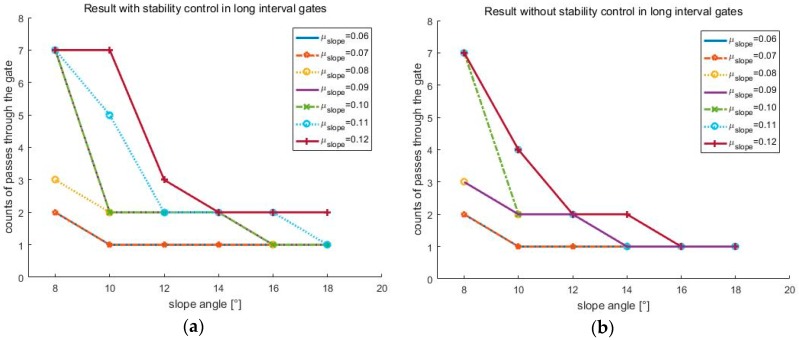
Algorithm performance according to friction coefficient and slope angle in long interval gates (**a**) with stability control; (**b**) without stability control.

First, Figure 13a is the lateral ZMP result tested by changing the friction coefficient $\mu_{slope}$ to 0.09. When the friction coefficient is lowered, the speed of the skiing robot is increased, and thus $F_{C}$ is increased. As $F_{C}$ increases, the ZMP becomes more likely to saturate. As a result, without the stability control being applied, the skiing robot fell down. Figure 13b shows the lateral ZMP result with a coefficient of friction of 0.12 and a slope angle of 10°. When the coefficient of friction was 0.1, even though the stability control was performed, it failed to pass through all of the gates when the slope angle was 10°. Thus, the test at the slope angle of 10° was conducted at the friction coefficient of 0.12. Under these conditions, Figure 14 shows the robot’s trajectory and Figure 15 shows the linear velocity of the robot. In Figure 14, the end of the blue line indicates the position of the overturn because of no control. This point corresponds to the point where the blue line, which means linear velocity, sharply in Figure 15. In addition to these cases, various simulations were conducted, and Figure 16 shows the results. Figure 16 shows the number of the gates passed out of a total of seven gates when the coefficient of friction and slope angle change. According to [6], the friction coefficient of the slope ranges from 0.02 to 0.2. At the friction coefficients of 0.02 and 0.2, the slope angles at which the robot could pass through all the gates were 2° and 15°, respectively.

## 6. Discussion

In this paper, the turning and stability control algorithms for skiing robots and a simulator for verifying them are proposed. This simulator is meaningful, in that it can be tested in various environments through simulations instead of a laborious experiment in an actual snow environment. In particular, in the real snow environment, various conditions, such as humidity and temperature, must be met, the appropriate slope angle should be found, and there should be no unwanted obstacles. Although it is difficult to achieve exactly the same turn radius as an actual experiment in the simulation, it can be used to quickly and easily verify ski algorithms in a variety of environments.

Many studies have dealt with the turn radius through experiments in real snow environments. However, the snow has very complex properties, so it is difficult to find the simulator that can accurately realize them. Even in the simulator Webots, which we used, it was difficult to realize the realistic snow itself. However, through researches of the forces that occur when skiing, we could apply the same force to the ski in the simulator and this can make us verify the proposed navigation and stability control algorithms without realizing snow.

Unlike traditional skis, carving skis are characterized by minimized skidding during a turn. In real snow, however, even with carving skis, skidding can occur, which results in a larger turn radius than expected. Nevertheless, this simulator has the advantage that the performance of the navigation and stability control algorithms of the robot can be estimated to some extent before the experiment on the real snow.

The navigation algorithm that was verified by this simulator was realized by LiDAR sensor. In [11,12,13,14], the gate was recognized by the camera. In this case, it was sensitive to light. If the light condition is changed, the gate may not be correctly recognized. In addition, the LiDAR sensor that was used in the simulation can scan from 0° to 180° in the forward direction, but the camera has a limited angle of view in most cases and it must undergo a complicated image processing process. Therefore, the LiDAR sensor does not need to be affected by the color of the gate, and it can make the robot move to the nearest gate through the distance to the gate that can be known in real-time.

However, even with such a navigation algorithm, it is impossible to properly follow the gate unless stability control is applied. In [10], the robot was prevented from falling by using posture control. However, by simply controlling the posture of the robot, it can easily fall when the slope change was abrupt or it moves with a tiny turn radius. On the other hand, if stability control is performed while using the ZMP that was measured by FSR sensors, it can be kept stable, even when the robot moves dynamically.

In real skiing, the original purpose of the carving turn is to keep the speed as far as possible when the skier turns. However, in the current robot skiing algorithm, the robot’s speed was reduced when stability control was applied. This is because the stability control gain was set high. In the proposed robot skiing algorithm, one of the main differences from real skiing is that there is no way for the robot to slow down, except for turning. However, if the robot’s speed continues to increase, the centrifugal force will increase, which makes it impossible to pass all the gates. Therefore, in this research, the stability control gain was set high to reduce the robot’s speed as much as possible when the robot turns.

The simulation results show the lateral ZMP, the robot’s trajectory, and the linear velocity. In the case of the short interval gates, the turning time is short, so the speed gradually increases. On the other hand, in the case of the long interval gates, the speed decreased during the turn, and the deceleration became larger when the control was performed. Therefore, in order to verify the performance of the algorithm, simulations were carried out with various friction coefficients and slope angles for the long interval gates that were more affected by stability control. As a result, it can be seen that the robot could pass through more gates in general when stability control was applied. However, this result also changed, depending on the positions of the gates and the point where the robot started. Therefore, even if the robot cannot pass all of the gates at a certain coefficient of friction and slope angle, it can pass through by adjusting the positions of the gates. The reason for this is that, if the robot turns less and sliding time is longer, the speed is increased greatly, reaching a situation where it cannot be controlled at all.

As a result, in future research, it is aimed to study the motions that can reduce the speed while the skiing robot is sliding, and the forces that are applied when the motions are taken. If the algorithm that the robot can control its speed is developed, it would be possible to decrease the stability control gain and increase the navigation control gain to make it more similar to the actual skiing. In addition, a new controller is needed to generate an optimal trajectory that the skiing robot can turn as fast as possible, without falling over. The method of designing this new controller remains as further research.

## 7. Conclusions

This study constructed a simulator environment that is similar to an actual ski slope and modeled the leaning angle of the body by inputting the desired turn radius. We also modeled the force that is required to move the robot in the input turn radius and implemented a gate passing algorithm while using a LiDAR sensor. In addition, the LiDAR sensor reduces the sensitivity to environmental changes as compared to using a camera to follow the gate and improves upon the stability by applying stability control through ZMP control. The ski simulation in the basic environment is now possible in the simulator; therefore, future work will be carried out in various environments, such as forming an irregular slope and changing the gate’s angle to create a more realistic environment. Finally, we will devise the motions to reduce the robot’s speed and a new controller to generate an optimal trajectory for the skiing robot. When this process has been completed, a simulator that can be used as a realistic environment before carrying out actual experiments will be completed.

## Figures and Tables

**Table 1:** Gate distances of the simulation.

Gate Number	Short Interval (m)		Long Interval (m)	
	Front	Horizon	Front	Horizon
1	13	1	13	2
2	21	5	23	12
3	29	4	33	3
4	37	8	43	14
5	49	6	58	2
6	61	9	70	11
7	73	11	85	4
8	85	7	-	-
